# Molecular Evolution, Structure, and Function of Peroxidasins

**DOI:** 10.1002/cbdv.201100438

**Published:** 2012-09-13

**Authors:** Monika Soudi, Marcel Zamocky, Christa Jakopitsch, Paul G Furtmüller, Christian Obinger

**Affiliations:** a) Department of Chemistry, Division of Biochemistry, Vienna Institute of BioTechnology at BOKU – University of Natural Resources and Life SciencesMuthgasse 18, A-1190 Vienna (phone: +43-1-476546073; fax: +43-1-476546059); b) Institute of Molecular Biology, Slovak Academy of SciencesDúbravská cesta 21, SK-84551 Bratislava

**Keywords:** Peroxidasin, Peroxidase, Immunoglobulin domain, Leucin-rich repeat domain, *von Willebrand* factor C

## Abstract

Peroxidasins represent the subfamily 2 of the peroxidase-cyclooxygenase superfamily and are closely related to chordata peroxidases (subfamily 1) and peroxinectins (subfamily 3). They are multidomain proteins containing a heme peroxidase domain with high homology to human lactoperoxidase that mediates one- and two-electron oxidation reactions. Additional domains of the secreted and glycosylated metalloproteins are type C-like immunoglobulin domains, typical leucine-rich repeats, as well as a *von Willebrand* factor C module. These are typical motifs of extracellular proteins that mediate protein–protein interactions. We have reconstructed the phylogeny of this new family of oxidoreductases and show the presence of four invertebrate clades as well as one vertebrate clade that includes also two different human representatives. The variability of domain assembly in the various clades was analyzed, as was the occurrence of relevant catalytic residues in the peroxidase domain based on the knowledge of catalysis of the mammalian homologues. Finally, the few reports on expression, localization, enzymatic activity, and physiological roles in the model organisms *Drosophila melanogaster, Caenorhabditis elegans*, and *Homo sapiens* are critically reviewed. Roles attributed to peroxidasins include antimicrobial defense, extracellular matrix formation, and consolidation at various developmental stages. Many research questions need to be solved in future, including detailed biochemical/physical studies and elucidation of the three dimensional structure of a model peroxidasin as well as the relation and interplay of the domains and the *in vivo* functions in various organisms including man.

## Introduction

Recently, we have reconstructed the phylogenetic relationships of the main evolutionary lines of the mammalian heme-containing peroxidases myelo-peroxidase (MPO), eosinophil peroxidase (EPO), lactoperoxidase (LPO), and thyroid peroxidase (TPO) [1]. Based on their occurrence and the fact that two main enzymatic activities are related to these metalloproteins, the peroxidase-cyclooxygenase superfamily was defined and shown to occur in all kingdoms of life [1]. Seven clearly separated subfamilies were found with chordata heme peroxidases comprising subfamily 1. Its representatives are involved in the innate immune system (MPO, EPO, and LPO) as well as hormone biosynthesis (TPO). Mature vertebrate peroxidases consist of only one (monomeric EPO and LPO) or two (homodimeric MPO and TPO) glycosylated, mainly α-helical domains with one autocatalytically modified heme per domain [2]. Well characterized chordata peroxidases evolved from multidomain enzymes called peroxidasins (subfamily 2) [1]. These peculiar proteins are found in vertebrates and invertebrates and have – in addition to a heme-containing domain of high homology with chordata peroxidases – flanking domains which are known to be important for protein–protein interaction or cell adhesion. These include leucine-rich regions, immunoglobulin-like domains, as well as a *von Willebrand* factor C (VWC) module.

In 1994, the first peroxidasin was described and found to occur in hemocytes of *Drosophila* [3]. Hemocytes are migratory cells present in the hemolymph of insects and are involved in (targeted) consolidation of extracellular matrix as well as in intracellular phagocytosis of apoptotic cells or foreign material (*e.g*., pathogens). In the following years, homologous genes were detected among numerous ecdysozoan [4] and almost all deuterostomian genomes [5] [6], implicating their importance in both invertebrate and vertebrate physiology. The human homologue of this subfamily, first cloned as a shorter mRNA fragment, was originally identified as a p53-responsive gene [7]. Finally, on a protein basis, the first human peroxidasin was detected in human colon cancer cells, but later also in squamous lung carcinoma cells, where it was initially named melanoma gene 50 (MG50) [8]. In the human genome, two different peroxidasins are encoded [1] and were originally designated as vascular peroxidase (VPO) 1 and 2 (VPO 2 is sometimes referred to as cardiac peroxidase), in relation to their high expression in the vascular system or heart [9]. However, both enzymes are multidomain proteins of subfamily 2 of the peroxidase-cyclooxygenase superfamily and thus should be named systematically human peroxidasin 1 (hsPxd01) and human peroxidasin 2 (hsPxd02). In recent years, several papers were published that report expression patterns [9–13], localization [9] [14], enzymatic activities [9], and physiological relevance of peroxidasins [15–18], but still many open questions remain regarding the relation between structure and function and the putative role(s) in the extracellular matrix formation and the innate immune system of invertebrates and vertebrates.

Here, we have reconstructed the molecular evolution of peroxidasins. We have analyzed the multidomain structure of the two main groups, *i.e*., invertebrate and vertebrate peroxidasins, and discuss the function of the enzymatic domain based on the knowledge about catalysis of human peroxidases [2]. Finally, we critically relate these facts to biochemical and biological features of these proteins.

## Results and Discussion

### Phylogenetic Reconstruction

**Fig. 1:**
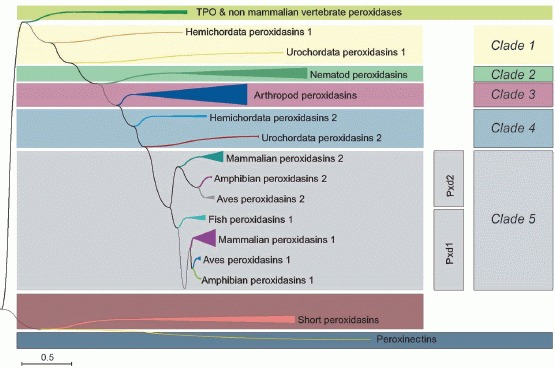
*Reconstructed unrooted phylogenetic tree of selected representatives of chordata peroxidases peroxinectins, and the peroxidasin subfamily, obtained by the neighbor joining* (NJ) *method*. For the reconstruction, the complete peroxidasin sequences encoding all domains were used. The abbreviations of the analyzed sequences are identical with those used in the *PeroxiBase* and are listed in the *Table*.

Genes encoding peroxidasins are found in vertebrates as well as in invertebrates. For the phylogenetic reconstruction, all available peroxidasin sequences (*i.e*., 106 in December 2011) were aligned and analyzed by three different methods. *Fig. 1* depicts the reconstructed unrooted tree obtained by the neighbor-joining (NJ) method; however, very similar trees were achieved by the minimum-evolution (ME) and maximum-likelihood (ML) methods. A clear separation into five peroxidasin clades is evident, with *Clades 1–4* representing invertebrate and *Clade 5* vertebrate oxidoreductases *(Fig. 1*). For the phylogenetic analysis, the full gene length including all domains was used; however, analyses based on only the peroxidase domain gave a very similar evolutionary tree (results not shown^1^).

Closely related with subfamily 3 (*i.e*., peroxinectins) of the peroxidase-cyclo-oxygenase superfamily [1], and thus somehow linking subfamilies 2 and 3, are short peroxidasins that lack all characteristic peroxidasin domains except the peroxidase domain. So far (December 2011), only eight genes are known and all occur in nematodes. Since these putative proteins do not show the typical multidomain structure of neither peroxinectins nor peroxidasins, they are not designated as a distinct clade. Echinozoan and ecdysozoan peroxinectins have integrin-binding motifs in addition to the peroxidase domain [1] [18] and, so far, no vertebrate representative was found.

**Fig. 2:**
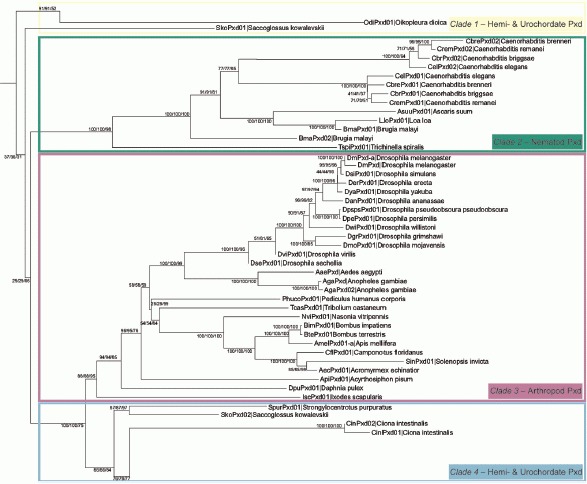
*Detail of the reconstructed phylogenetic tree of peroxidasins showing invertebrate peroxidasin evolution (Clades 1-4)*. Bootstrap values in the nodes were obtained from neighbor-joining (NJ), minimum-evolution (ME), and minimum-likelihood (ML) methods, respectively.

At the origin of evolution (*Clade 1*) of multidomain peroxidasins, proteins from hemichordates and urochordates are found, which represent the closest neighbors of chordata peroxidases (*Fig. 1*). Clearly separated from *Clade 1* are nematode peroxidasins (*Clade 2*), arthropod peroxidasins (*Clade 3*), and a second group of hemichordate and urochordate proteins (*Clade 4; Fig. 2*).

**Fig. 3:**
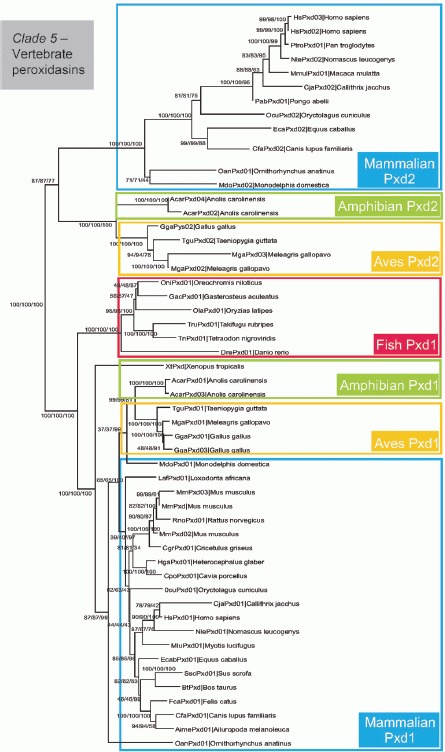
*Detail of the reconstructed phylogenetic tree of peroxidasin evolution showing vertebrate peroxidasins (Clade 5*). Bootstrap values in the nodes were obtained from NJ, ME, and ML methods, respectively.

Vertebrate peroxidasins (*Clade 5*) can be divided into two branches, namely peroxidasins 1 (Pxd1) and peroxidasins 2 (Pxd2), sometimes referred as peroxidasin-like proteins. In each branch, a clear segregation in fish, amphibian, bird, and mammalian proteins is obvious (*Fig. 3*). In the human genome, two peroxidasins are encoded that share a sequence identity of 63%. Peroxidasin 1 from *Homo sapiens* (hsPxd01) is located on chromosome 2p25, whereas human peroxidasin 2 (hsPxd02) is located on chromosome 8q11 [9]. A closer look to *Fig. 3* depicts the presence of a third human peroxidasin, which is almost identical to hsPxd02 – except for five amino acids – and might be a splicing variant.

### Domain Assembly and Architecture

**Fig. 4:**
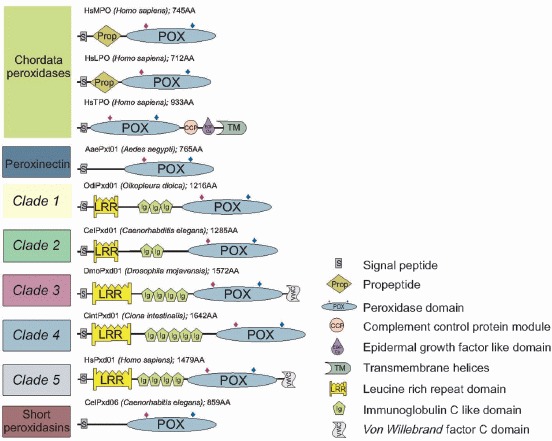
*Schematic presentation of subunit assembly of representatives of the chordata heme peroxidases, peroxinectins, short peroxidasins, and the five clades of the peroxidasin subfamily*. The domain architectures were adopted from the *Pfam-Database* at the *Sanger Institute* (http://www.sanger.ac.uk) and *Prosite Database* at *ExPASy*, the *SIB Bioinformatics Resource Portal* (http://prosite.expasy.org). The rhombuses indicate the position of the distal and proximal histidine in the peroxidase domain.

So far, no three-dimensional structure of a peroxidasin is known, but comparative sequence analysis and secondary-structure prediction clearly indicate the presence of four different domains of distinct assembly and architecture. *Fig. 4* shows the domain assembly in invertebrate and vertebrate peroxidasins. In the latter, and thus also in both human peroxidasins, the primary transcript has a signal peptide for extracellular secretion followed by five leucine-rich repeats with N-terminal and C-terminal capping motifs, four successive immunoglobulin domains, one heme-binding peroxidase domain, and a C-terminal *von Willebrand* factor C module (*Fig. 4*).

*Clade 1* and nematode peroxidasins (*Clade 2*) vary in the number of both the leucine-rich repeats as well as immunoglobulin motifs. Additionally, they do not contain a C-terminal *von Willebrand* factor C, suggesting that this feature is phylogenetically the newest addition to these multidomain proteins. Arthropod peroxidasins (*Clade 3*) also show variable numbers of leucine-rich repeats and immunoglobulin domains, but the majority of the putative proteins seem to contain already a C-terminal *von Willebrand* factor C, whereas in the second group of hemichordate and urochordate peroxidases (*Clade 4*) the *von Willebrand* factor C is absent.

Up to now, we have no detailed knowledge about the relation between domain assembly and structure and their physiological relevance in peroxidasins. The only enzymatic domain is the heme-containing peroxidase domain and its sequence is critically analyzed below. All the other domains are found in thousands of proteins in various contexts.

### Leucine-Rich Repeats

Leucine-rich repeats (LRR) occur in numerous proteins and all of them appear to be involved in protein–protein interactions including cell adhesion and signal transduction, extracellular matrix assembly, platelet aggregation, neuronal development, RNA processing, and immune response [19]. A striking example for the functional variety of LRRs is the adaptive immune system of jawless fishes. They possess variable lymphocyte receptors (VLR) built from proteins that are completely unrelated to immunoglobulins. Interestingly, they are reported to be generated from one or two germ lines of VLR genes by genomic rearrangement of flanking LRR cassettes [20].

Seven subclasses of LRR motifs differing in length and consensus sequence of the variable segments (typical, RI-like, CC, PS, SDS22-like, bacterial, and TpLRR) have been proposed [21]. In peroxidasins, only typical LRR cassettes are found. This motif exhibits a solenoid structure where each repeat represents a turn that is typically 20–30 amino acids long. It contains a conserved eleven-residue hallmark sequence (LxxLxLxxNxL) corresponding to a β-sheet that forms the concave side of the solenoid [19]. By contrast, the convex side shows a greater variability and may consist of α-helical structures including *3*_10_ helices, polyproline II conformation, and β-turns depending on the amino acid composition and length of the variable segment [22]. The conserved leucines as well as other mainly hydrophobic amino acids are arranged to a characteristic tightly packed structure forming the inner core of the solenoid and contribute to the stability of the LRR domain. Many ligand–LRR protein complexes depict the ligand more or less surrounded by the concave surface of the parallel β-sheets of the LRR domain, however, ligand interactions with the convex side have also been observed. Generally, ligand binding does not induce major rearrangements in the LRR structure, since the free and bound forms superimpose [19].

**Fig. 5:**
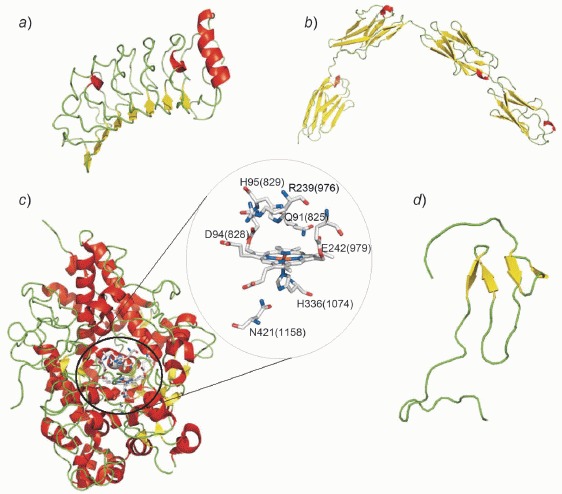
*Three-dimensional structures of domains of human peroxidasin 1* (hsPxd01). *a*) Model of leucine-rich repeat (LRR) domain with N- and C-terminal capping motifs. *b*) Model of C-like immunoglobulin domain (Ig). c) Overall structure of homologous goat LPO (PDB code 2R5L) with detailed presentation of catalytic active site residues. Numbering corresponds to the mature LPO, whereas numbering in parentheses corresponds to full length hsPxd01, respectively. *d*) Model of *von Willebrand* factor C (VWC). All models have been calculated using the ESyPred3D server.

The hydrophobic core of the LRR domains may be protected from opening by N-terminal and C-terminal cysteine-rich flanking regions, which are often found in extracellular LRR proteins including peroxidasins [21]. The N-terminal capping motif (LRRNT) is described as a single β-strand, antiparallel to the main β-sheet, followed by a short LRR of 20 or 21 residues, whereas the C-terminal capping motif (LRRCT) possesses an a-helix covering the hydrophobic core of the last LRR [21]. *Fig. 5, a*, shows a model of the LRR domain of hsPxd01 that is built from five tandem typical LRRs framed by LRRNT and LRRCT.

### Immunoglobulin Domains

Since peroxidasins in addition contain immunoglobulin (Ig) domains, they are also accounted to the heterogenic immunoglobulin superfamily (IgSF). This major group of proteins differs in tissue distribution, amino acid composition, and biological role, but all representatives possess at least one structurally discrete domain of *ca*. 100 amino acids exhibiting the Ig-fold [23]. The differentiation of immunoglobulin domains is based on the composition of the two β-sheets that form the sandwich-like fold. The constant domains (C-domains) are comprised of seven strands and the variable domains (V-domains) of eight, nine, or ten strands [24]. The standardized *IMGT (International ImMunoGeneTics Information System*) discriminates immunoglobulin domains according to following categories: C-domains (including immunoglobulins and T-cell receptors of all jawed vertebrates), C-like domains (proteins other than immunoglobulins or T-cell receptors), V-domains (immunoglobulins and T-cell receptors with V-J-region and V-D-J-region) and V-like domains (proteins other than immunoglobulins or T-cell receptor with V-J-region and V-D-J-region) [25]. According to this systematics, peroxidasins have C-like Ig-domains.

As mentioned before, the Ig-fold is a wide-spread protein motif with various biological functions, although the common feature seems to be cell adhesion and pattern recognition. An example for a structural function of immunoglobulin domains are the Ig-tandems of titin, a myofilament protein and the largest mammalian protein. *Von Castelmur et al*. [26] determined the crystal structure of a fragment from the skeletal I-band of soleus titin. This structure was also used as a template for tertiary-structure modelling of the Ig-domains of hsPxd01 (*Fig. 5*, *b*).

### *Von Willebrand* Factor Type C

The C-terminal *von Willebrand* factor type C (VWC) module is also referred as chordin-like, cysteine-rich (CR) repeat. It contains *ca*. 60–80 amino acids and is defined by a consensus sequence of ten cysteines [27]. The name originates from the five types of structural domains comprising the *von Willebrand* factor (VWF), a multimeric blood glycoprotein that binds and stabilizes clotting factor VIII and mediates platelet adhesion [28]. This motif has been identified in more than 500 extracellular matrix proteins including CCN (cysteine-rich protein 61, connective tissue growth factor proteins, nephroblastoma overexpressed gene), procollagen, thrombospondin, glycosylated mucins, and neuralins with varying copy numbers [29]. Peroxidasins contain only one C-terminal copy, similar to the CCN proteins.

For most VWC modules, the cellular role has still be to investigated [29], however, binding and regulating bone morphogenetic proteins (BMPs) and transforming the tissue growth factor beta (TGF-β) are the two most common functions attributed to the VWC domain [30]. Moreover, the VWC domain might be involved in oligomerization of proteins [28].

The three-dimensional structure of a prototypical chordin-like, cysteine-rich repeat from collagen IIAwas determined by *O'Leary et al*. [27]. They found that the VWC domain exhibits a two-subdomain architecture connected *via* a short linker region. A model of the VWC domain of hsPxd01 is shown in *Fig. 5*, *d*. The C-terminal subdomain adopts a rather irregular and flexible structure, whereas the N-terminal subdomain of peroxidasin consists of two double stranded antiparallel β-sheets, in contrast to the N-terminal subdomain of collagen IIA that contains one double-stranded and one triple-stranded antiparallel β-sheet. Interestingly, there is a structural similarity between the N-terminal subdomain of the VWC module and the fibronectin type 1 (FN1) domain. Since FN1 domains are only found in vertebrates, in contrast to VWCs, which have been identified in all eukaryotes, an evolutionary relationship between these two domains might be possible [27].

### The Enzymatic Heme Domain

**Fig. 6:**
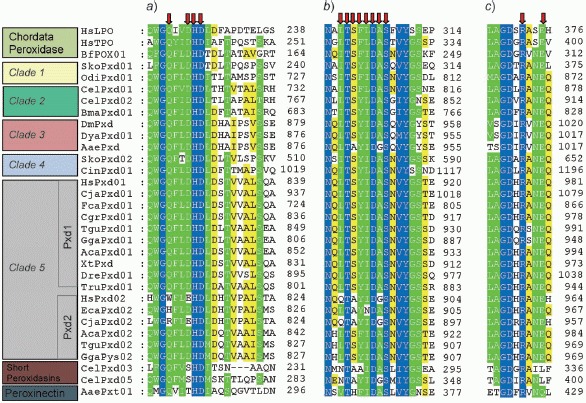
*Selected parts of the multiple sequence alignment of 32 sequences from members of the peroxidasin subfamily as well as representatives of chordata peroxidases, peroxinectins, and short peroxidasins*. Catalytically important residues of the distal heme cavity are highlighted by arrows. *a*) Area around the essential distal Asp-His-Asp as well as Gln (compare with *Fig. 5*, *c*). *b*) Sequence area around the known Ca2^+^-binding site in mammalian peroxidases. *c*) Area around the catalytic distal Arg and Glu, the latter being involved in heme to protein ester linkage in mammalian peroxidases.

**Fig. 7:**
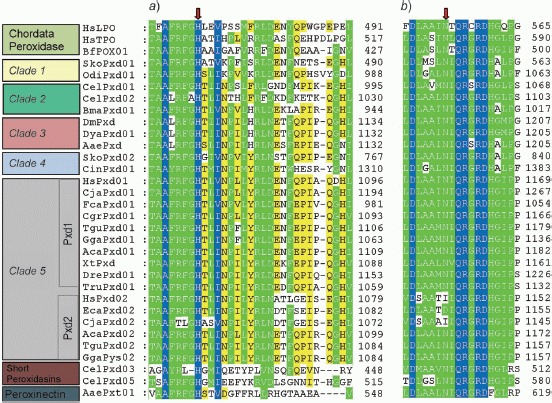
*Selected parts of the multiple sequence alignment of 32 sequences from members of the peroxidasin subfamily as well as representatives of chordata peroxidases, peroxinectins, and short peroxidasins*. Catalytically important residues of the proximal heme cavity are depicted. Sequence areas around *a*) the proximal histidine and *b*) its H-bonding partner asparagine are shown.

Subfamilies 1–3 of the peroxidase-cyclooxygenase superfamily contain one enzymatic peroxidase domain [1]. Multiple sequence alignment reveals highly conserved regions (*Figs. 6* and *7*). They correspond to functionally and structurally essential motifs, as known from mammalian peroxidases. The secondary structure of mammalian peroxidases as well as of the peroxidase domain of peroxidasins is predominantly a-helical with a central heme-containing core composed of five helices (*Fig. 5*, *c*).

Both the distal and proximal histidines in the heme cavity as well as the corresponding H-bonding partners are located within α-helices. Essential distal residues in mammalian peroxidases are Gln91, His95, and Arg239 (*Fig. 5*, *c*; numbering corresponds to mature lactoperoxidase and numbering in parentheses corresponds to hsPxd01). The peroxidase-typical distal pair His-Arg is found in all heme peroxidases from both superfamilies (peroxidase-cyclooxygenase and peroxidase-catalase super-family) [1] and is also fully conserved in all peroxidasins (*Fig. 6*). This pair is important in the heterolytic cleavage of H_2_O_2_ [2]. In mammalian peroxidases, Gln91 (*Fig. 5*, *c*) is involved in the maintenance of the distal H-bond network as well as halide binding [2]. This essential glutamine, which is part of a conserved motif ‘W/F-G-Q-F’, is present in almost all analyzed peroxidasin sequences, except short peroxidasins and group Pxd2 of *Clade 5* enzymes including hsPxd02 (*Fig. 6*).

The second adjacent conserved motif in mammalian peroxidases, which is also fully conserved in peroxidasins and includes the distal His95, is ‘Asp-His-Asp’. Asp94 is known to be involved in ester-bond formation with the OHCH_2_ group at C(5) of pyrrole ring C of the heme (*Fig. 5*, *c*), and it is found in all peroxidasins, except group Pxd2 of *Clade 5* and short peroxidasins. Regarding Asp96, its role as a ligand of Ca2^+^ is well established. The role of the distal Ca2^+^-binding site could be to stabilize both the distal heme cavity architecture as well as to mediate the assembly of the mature peroxidases [2]. In both lactoperoxidase and myeloperoxidase, the Ca2^+^-binding site has a typical pentagonal bipyramidal coordination geometry [2] and the cation is liganded by several highly conserved residues. One is the already mentioned Asp96 and the other ligands are part of a loop that consists of eight residues, *i.e.*, ‘Leu-Thr-Ser-Phe-Val-Asp-Ala-Ser’ (amino acid number 333–340 of the mature LPO), and is found in all chordata peroxidases. This motif is also present in all peroxidasins, although some mutations, especially in short peroxidasins and group Pxd2 of *Clade 5*, are observable.

All mammalian peroxidases have their prosthetic group covalently bound to the protein and, besides Asp94 mentioned above, a conserved Glu242 forms the second ester bond. Except short peroxidasins, all other members of this protein family have this glutamate residue fully conserved (*Fig. 6*).

Structurally and functionally important motifs on the proximal side of mammalian peroxidases include the proximal histidine (His336) and its H-bonding partner asparagine (Asn421; *Fig. 5*, *c*). Both residues govern the heme-iron reactivity by controlling the electron density at the metal [2]. Inspection of *Fig. 7* again demonstrates that *Clades 1–4* and group Pxd1 of *Clade 5* are chordata peroxidase-like, whereas in group Pxd2 of vertebrate peroxidasins variable amino acids (Ile, Asp, Asn) are found at the corresponding position of Asn421.

Summing up, sequence analysis of the enzymatic domain of peroxidasins suggests a similar function as in vertebrate peroxidases. All catalytically relevant residues (with exception of the MPO-specific methionine that is responsible for the third covalent linkage between the autocatalytically modified heme and the protein) are also found in the peroxidase domain of most of the peroxidasins. This suggests similar enzymatic features, including catalysis of one- and two-electron oxidation reactions, and some preliminary biochemical data support this observation [9] [14], although the reported activities (e.g., tyrosine and halide oxidation) seem to be significantly lower compared to mammalian peroxidases. Only short peroxidasins and group Pxd2 of vertebrate peroxidases (including hsPxd02) show alternative amino acids at structurally and functionally important positions, and it is not clear at the moment whether these enzymes are functionally comparable with peroxidases.

### Physiological Roles

So far, only a handful of studies have been published that might give an indication of the *in vivo* function of peroxidasins. Focusing on the enzymatic domain only, one could speculate about similar biological roles as reported for the closely related and homologous chordata peroxidases including MPO, EPO, and LPO. The latter are involved in innate immune-defense reactions against invading pathogens and are stored in high concentration in granules of neutrophilic (MPO) or eosinophilic leukocytes (EPO) that are recruited to sites of pathogen invasion and inflammation [31] [32], whereas secreted lactoperoxidase acts as extracellular bactericidal agent in exocrine secretions [33]. These peroxidases catalyze the production of hypohalous acids or hypothiocyanate from H_2_O_2_ and (pseudo-) halide anions, but also act as efficient one-electron oxidants [2].

Another physiological role of chordata peroxidases is cell adhesion and formation of extracellular matrix (ECM). It has been demonstrated that the extracellular oxidants generated by cationic mammalian peroxidases (*e.g*., MPO) can have both damaging and protective effects on the ECM, including inactivation of metalloproteinases [34] [35] and formation of dityrosine crosslinks [36].

Among peroxidase-domain containing proteins of the same superfamily, also peroxinectins (subfamily 3 of the peroxidase-cyclooxygenase superfamily) might give some indications about the functionality of peroxidasins, although from this subfamily (that has no vertebrate representative) only very few reports can be found in the literature. These metalloenzymes seem to be involved in the promotion of cell–ECM adhesion via their integrin-binding motifs [37]. Interestingly, also MPO has been shown to promote integrin-mediated adhesion of neutrophils [38].

Regarding the overall structure and size as well as domain assembly, peroxidasins are the most complex representatives of peroxidase-domain-containing enzymes of the peroxidase-cyclooxygenase superfamily. Sequence analysis clearly suggests an enzymatic functionality similar to LPO and EPO (except in short peroxidasins and group Pxd2 of *Clade 5*). The motifs typical of extracellular proteins (Igs, LRRs, and VWC) might contribute to association with ECM. Both the roles in innate immune defense and extracellular matrix consolidation are often addressed in various contexts in the literature.

As already mentioned, the first peroxidasin was identified in 1994 in *Drosophila melanogaster* (*Clade 3* peroxidasins; *Fig. 2*) and has shown to be associated with the function of insect hemocytes and plasmatocytes [3] [7]. Similar to neutrophils, hemocytes are migratory cells that phagocytose foreign and dead cells and deposit ECM. Peroxidasin expression in *Drosophila* is widely used as a molecular marker for the early hemocyte lineage [39], and hemocytes are crucial for a morphogenetic event known as condensation of the ventral nerve cord (VNC). If hemocyte migration is blocked, deposition of ECM components, including peroxidasin and type IV collagen, does not occur, leading to failure of ventral nerve cord condensation [40]. *Drosophila* peroxidasin was reported to be a homotrimer that catalyzes H_2_O_2_-mediated tyrosine and iodide oxidation [3].

The second invertebrate model organism in which the *in vivo* role of peroxidasin was studied is *Caenorhabditis elegans* [15]. Two orthologs are found in *C. elegans* (CelPxd01 and CelPxd02; *Fig. 2*) with apparently antagonistic functions. CelPxd02 was identified upon screening for mutants defective in embryonic worm development. It was found to be essential for specific stages of morphogenesis and epidermal muscle attachment as well as postembryonically for basement membrane integrity [15]. The peroxidase activity (which – based on sequence analysis – should be LPO-like) was shown to be responsible for these developmental roles. In adult worms, loss of CelPxd02 promotes regrowth of axons after injury, providing evidence that *C. elegans* extracellular matrix can play an inhibitory role in axon regeneration. By contrast, loss of CelPxd01 did not cause developmental effects. Moreover, CelPxd02 mutant phenotypes were suppressed by loss of function of CelPxd01 and exacerbated by its overexpression, suggesting antagonistic roles.

The first study on a (non-mammalian) vertebrate peroxidasin was performed on group 1 peroxidasin (*Fig. 3*) from *Xenopus tropicalis* in 2005 [10]. This representative is expressed in several distinct tissues during early development, including the neural tube and the tail-forming region, and has also a role in modifying extracellular matrix components necessary in morphogenesis, again suggesting a role of the peroxidase domain in catalyzing one-electron oxidation reactions.

Investigations on human peroxidasins started in 1999 [7]. It was demonstrated that besides the full-length mRNA of hsPxd01, also a shorter 4.5 kb mRNA version occurs in human colon EB1 cancer cells undergoing p53-dependent apoptosis. Characterization of the smaller mRNA showed that the N-terminal part of hsPxd01 including the signal peptide was not present, whereas the C-terminal portion including the peroxidase domain was present. The authors hypothesized that the protein missing a signal sequence would accumulate in the cytoplasm, and since the peroxidase domain is intact, it could potentially increase the cellular production of reactive oxygen species (ROS), which are powerful inducers of apoptosis by inactivation of the p53 tumor suppressor protein [7]. Based on the knowledge of catalysis of homologous mammalian peroxidases [2], the effect on p53 could be mediated by hypohalous acids or hypothiocyanate released from peroxidasin rather than by activated oxygen species, since peroxidases consume H_2_O_2_ and do not release O_2_^•−^ at reasonable rates.

Finally, the cloning of human and mice peroxidasins and initial characterization of hsPxd01 were reported [9]. Heme-containing hsPxd01was recombinantly expressed in HEK cells, and the prosthetic group of hsPxd01 was shown to be covalently attached to the protein *via* two ester bonds very similar to LPO or EPO [9]. The protein was shown to exhibit both peroxidase activity (tetramethylbenzidine oxidation) as well as halogenation activity (two-electron oxidation of Cl^−^). However, the reported activities were very low compared to mammalian peroxidases. The finding of chlorination activity at neutral pH, which so far was only attributed to MPO, is peculiar, since hsPxd01 cannot form the MPO-typical (e^−^ withdrawing) sulfonium-ion linkage and thus this reactivity must be addressed in future biochemical studies.

Based on its halogenation activity and localization, the role of hsPxd01 in low-density lipoprotein oxidation and endothelial cell apoptosis was investigated [17]. It was demonstrated that expression of hsPxd01 in endothelial cells correlates with LDL oxidation in a time- and concentration-dependent manner. Activity of hsPxd01 was shown to be dependent on NADPH-oxidase activity, which initially forms O_2_^•−^ that dismutates to H_2_O_2_ necessary to initiate both peroxidase and halogenation activity.

*Peterfi et al*. [14] demonstrated the secretion of human hsPxd01 as well as the formation of peroxidasin-containing, fibril-like structures by differentiated myofibroblasts. Since myofibroblasts appear during wound healing, it is tempting to speculate that the peroxidase domain functions in an antimicrobial manner, whereas the other domains could participate in the ECM formation. Stabilization of the ECM could also be achieved by the enzymatic formation of dityrosine crosslinks.

In another study [18], homozygous mutations in the gene encoding hsPxd01were associated with a spectrum of ocular anterior segment dysgenesis phenotypes. Although the precise effect of these mutations is unknown, the pathogenic impact is obvious. Finally, it was demonstrated that hsPxd01 is also expressed in various cancer cells, including breast cancer, melanoma, colon cancer, and metastatic gilomas [8] [11] [12]. Hence, hsPxd01 seems to be a signature gene of heme oxygenase-1 (HO-1) [12], an enzyme associated with tumor angiogenesis. A loss of the adhesion promoting effect of HO-1 in hsPxd01-silenced cells was observed. This suggests also a role of hsPxd01 in cell adhesion and invasion as well as in the transition of benign tumors to invasive and malignant cancers [12].

Summing up, peroxidasins are multidomain heme peroxidases with a substrate spectrum similar to mammalian peroxidases. Based on sequence analysis and reported biochemical data, they perform one-electron oxidation reactions including tyrosine oxidation as well as two-electron oxidation reactions of (pseudo-) halides. Hypochlorous acid formation is questionable and must be addressed in future investigations. In any case, physiological studies have shown that the enzymatic domain is essential for the *in vivo* activity and might contribute to extracellular matrix consolidation as well as antimicrobial defense. The additional domains (Igs, LRRs, and VWC) are typical for extracellular proteins and may help in targeting the peroxidase to its site of action. However, a distinct function of the individual domains and their relation to the reported *in vivo* roles has not been demonstrated so far. Clearly, more detailed biochemical and biophysical studies as well as the elucidation of the three-dimensional structure of peroxidasins are necessary. These studies must include invertebrate and both groups of vertebrate peroxidasins, since – as sequence analysis suggests – they most probably differ in catalysis and, in consequence, in function.

This project was supported by the *Austrian Science Fund* and the doctoral program *BioToP – Biomolecular Technology of Proteins* (FWF W1224).

## Experimental Part

### Data Mining

All currently (December 2011) available 106 peroxidasin sequences were collected from public databases (*Uniprot, NCBI, PeroxiBase, Ensembl*). Selected representatives of non-mammalian vertebrate peroxidase (BbePOX01 *Branchiostoma floridae*, BbePOX01 *Branchiostoma belcheri*, HrPOX *Halocynthia roretzi*), HsTPO Human thyroid peroxidase, and *Aedes aegypti* peroxinectin (AaePxt01) sequences were retrieved from the *PeroxiBase* database [41] and used as an outgroup. Sequence data are given in the *Table*.

### Multiple Sequence Alignment

A multiple sequence alignment of the 112 enzymes was constructed with ClustalW [42] with following parameters: for pairwise alignment, gap opening penalty 9 and gap extension penalty 0.1; for multiple alignment, gap opening penalty 8 and gap extension penalty 0.2. *Gonnet* protein weight matrix was used and the gap separation distance was set to 4. The delay divergent cut off was defined with 25%. The output was displayed with Genedoc [43].

### Phylogeny Reconstruction

*Distance Method*. The multiple sequence alignment of the 112 selected enzymes was subjected to the neighbor-joining (NJ) method of the MEGA5 package [44] with the *Jones–Taylor–Thornton* (JTT) model of amino acid substitution, pairwise deletion of gaps, and homogenous pattern among lineages. The test of phylogeny was conducted with the bootstrap method, by setting the bootstrap replications to 1000. The optimized γ parameter for this set was determined as γ = 1.75.

**Table:** Table Abbreviations of the 106 Peroxidasin Sequences Used in This Study

Name	Entry IDa)	Organism	Lenght (aa)
AecPxd01	10036	*Acromyrmex echinatior*	1305
ApiPxd01	7638	*Acyrthosiphon pisum*	1669
AaePxd	3368	*Aedes aegypti*	1528
AcarPxd01	-	*Anolis carolinensis*	1482
AcarPxd02	-	*Anolis carolinensis*	1422
AcarPxd03	-	*Anolis carolinensis*	1436
AcarPxd04	-	*Anolis carolinensis*	1374
AgaPxd	4043	*Anopheles gambiae*	1559
AgaPxd02	7639	*Anopheles gambiae*	1514
AimePxd01	7610	*Ailuropoda melanoleuca*	1466
AmelPxd01-a	3671	*Apis mellifera*	1293
AsuuPxd01	-	*Ascaris suum*	1259
BmalPxd02	7644	*Brugia malayi*	999
BmalPxd01	7642	*Brugia malayi*	1149
BimPxd01	-	*Bombus impatiens*	1290
BtePxd01	-	*Bombus terrestris*	1290
BtPxd	3356	*Bos taurus*	1475
CbrePxd01		*Caenorhabditis brenneri*	1280
CbrePxd02	-	*Caenorhabditis brenneri*	1382
CbrPxd01	4289	*Caenorhabditis briggsae*	1288
CbrPxd02	4288	*Caenorhabditis briggsae*	1355
CbrPxd03b)	4287	*Caenorhabditis briggsae*	658
CbrPxd04b)	4286	*Caenorhabditis briggsae*	728
CbrPxd05b)	4285	*Caenorhabditis briggsae*	724
CbrPxd06b)	4284	*Caenorhabditis briggsae*	864
CelPxd01	3359	*Caenorhabditis elegans*	1285
CelPxd02	4103	*Caenorhabditis elegans*	1328
CelPxd03b)	4139	*Caenorhabditis elegans*	655
CelPxd04b)	4142	*Caenorhabditis elegans*	729
CelPxd05b)	4141	*Caenorhabditis elegans*	724
CelPxd06b)	4140	*Caenorhabditis elegans*	859
CremPxd01	-	*Caenorhabditis remanei*	1317
CremPxd02	-	*Caenorhabditis remanei*	1360
CflPxd01	7646	*Camponotus floridanus*	1303
CfaPxd01	10035	*Canis lupus familiaris*	1588
CfaPxd02	3358	*Canis lupus familiaris*	1429
CjaPxd01	7609	*Callithrix jacchus*	1577
CjaPxd02	7617	*Callithrix jacchus*	1456
CpoPxd01	-	*Cavia porcellus*	1479
CgrPxd01	-	*Cricetulus griseus*	1475
CintPxd01	7647	*Ciona intestinalis*	1642
CintPxd02	-	*Ciona intestinalis*	1279
DrPxd01	7648	*Danio rerio*	1530
DpulPxd01	-	*Daphnia pulex*	1262
DanPxd01	7649	*Drosophila ananassae*	1531
DerPxd01	7650	*Drosophila erecta*	1526
DgrPxd01	7651	*Drosophila grimshawi*	1534
DmPxd	3370	*Drosophila melanogaster*	1535
DmPxd-a	3369	*Drosophila melanogaster*	1527
DmoPxd01	7652	*Drosophila mojavensis*	1572
DpePxd01	7653	*Drosophila persimilis*	1534
DpspsPxd01	7654	*Drosophila pseudoobscura pseudoobscura*	1529
DsePxd01	7655	*Drosophila sechellia*	880
DsiPxd01	7656	*Drosophila simulans*	1528
DviPxd01	7657	*Drosophila virilis*	892
DwiPxd01	7658	*Drosophila willistoni*	1540
DyaPxd01	7659	*Drosophila yakuba*	1528
EcabPxd01	5825	*Equus caballus*	1431
EcabPxd02	7625	*Equus caballus*	1466
FcaPxd01	7692	*Felis catus*	1293
GgaPxd01	4049	*Gallus gallus*	1456
GgaPxd02	7673	*Gallus gallus*	1338
GacPxd01	7682	*Gasterosteus aculeatus*	1440
HglPxd01	-	*Heterocephalus glaber*	1412
HsPxd01	3355	*Homo sapiens*	1479
HsPxd02	5398	*Homo sapiens*	1463
HsPxd03	5827	*Homo sapiens*	1463
IsPxd01	7663	*Ixodes scapularis*	1111
LloPxd01	-	*Loa loa*	1236
LafPxd01	-	*Loxodonta africana*	1475
MmulPxd01	7616	*Macaca mulatta*	1413
MgaPxd01	-	*Meleagris gallopavo*	1459
MgaPxd02	-	*Meleagris gallopavo*	1342
MgaPxd03	-	*Meleagris gallopavo*	1374
MdoPxd01	5814	*Monodelphis domestica*	1627
MdoPxd02	5830	*Monodelphis domestica*	1542
MmPxd01	3357	*Mus musculus*	1475
MmPxd02	7680	*Mus musculus*	1431
MmPxd03	7681	*Mus musculus*	1379
MluPxd01	-	*Myotis lucifugus*	1133
NviPxd01	7676	*Nasonia vitripennis*	1240
NlePxd01	-	*Nomascus leucogenys*	1747
NlePxd02	-	*Nomascus leucogenys*	1463
OdiPxd01	-	*Oikopleura dioica*	1216
OanPxd01	5826	*Ornithorhynchus anatinus*	1469
OanPxd02	7674	*Ornithorhynchus anatinus*	1218
OniPxd01	-	*Oreochromis niloticus*	1462
OcuPxd01	7637	*Oryctolagus cuniculus*	1411
OcuPxd02	7624	*Oryctolagus cuniculus*	1467
OlaPxd01	7686	*Oryzias latipes*	1483
PtroPxd01	5828	*Pan troglodytes*	1463
PhucoPxd01	7677	*Pediculus humanus corporis*	1266
PabePxd01	7623	*Pongo abelii*	1300
RnoPxd01	7664	*Rattus norvegicus*	1475
SkoPxd01	7615	*Saccoglossus kowalevskii*	794
SkoPxd02	7622	*Saccoglossus kowalevskii*	1055
SinPxd01	-	*Solenopsis invicta*	1299
SpurPxd01	4101	*Strongylocentrotus purpuratus*	1421
SscPxd01	-	*Sus scrofa*	1479
TguPxd01	7678	*Taeniopygia guttata*	1490
TguPxd02	7679	*Taeniopygia guttata*	1475
TspiPxdOl	-	*Trichinella spiralis*	1276
TrubPxd01	7685	*Takifugu rubripes*	1434
TnPxd01	7618	*Tetraodon nigroviridis*	1379
TcasPxd01	4102	*Tribolium castaneum*	1388
XtPxd	4051	*Xenopus tropicalis*	1460

a) Entry ID in the *PeroxiBase* (http://peroxibase.toulouse.inra.fr/).

b) Member of the short peroxidasins.

### Minimum-Evolution (*ME*) Method

The phylogeny was also reconstructed using the ME method of the MEGA5 package [44] with following options: JTT model; bootstrap replications 1000; γ set to 1.75; close-neighbor interchange (CNI) was chosen as heuristic model and search level 2; the initial tree was obtained by NJ; homogenous pattern among lineages and pairwise deletion for gaps/missing data treatment.

### Maximum-Likelihood (*ML*) Trees

Additionally, the ML trees were inferred with the MEGA5 package [44] by using the JTT model, the γ parameter set to 3, and with 100 bootstrap replications; CNI and all sites were used for gaps/missing data treatment.

Protein secondary and tertiary structure prediction of the various domains of peroxidasin was performed with PSIPRED [45] and EsyPred3D [46].

## Footnotes

1) A supplementary figure showing the unrooted phylogenetic tree reconstructed with only the catalytic peroxidase domain is available upon request from the author.

## References

[1] Zamocky M, Jakopitsch C, Furtmüller PG, Dunand C, Obinger C (2008). Proteins: Struct., Funct., Bioinf.

[2] Furtmüller PG, Zederbauer M, Jantschko W, Helm J, Bogner M, Jakopitsch C, Obinger C (2006). Arch. Biochem. Biophys.

[3] Nelson RE, Fessler LI, Takagi Y, Blumberg B, Keene DR, Olson PF, Parker C, Fessler JH (1994). EMBO J.

[4] Scott AL, Ghedin E (2009). Parasitol. Int.

[5] Okazaki N, Kikuno R, Ohara I, Inamoto S, Aizawa H, Yuasa S, Nakajima D, Nagase T, Ohara O, Koga H (2003). DNA Res.

[6] Team MammalianGeneCollection(MGC)Program (2002). Proc. Natl. Acad. Sci. U.S.A.

[7] Horikoshi N, Cong J, Kley N, Shenk T (1999). Biochem. Biophys. Res. Commun.

[8] Mitchell MS, Kan-Mitchell J, Minev B, Edman C, Deans RJ (2000). Cancer Res.

[9] Cheng G, Salerno JC, Cao Z, Pagano PJ, Lambeth J (2008). Free Radical Biol. Med.

[10] Tindall AJ, Pownall ME, Morris ID, Isaacs HV (2005). Dev. Dyn.

[11] Liu Y, Carson-Walter EB, Cooper A, Winans BN, Johnson MD, Walter KA (2010). J. Neurooncol.

[12] Tauber S, Jais A, Jeitler M, Haider S, Husa J, Lindroos J, Knöfler M, Mayerhofer M, Pehamberger H, Wagner O, Bilban M (2010). Mol. Cancer.

[13] Homma S, Shimada T, Hikake T, Yaginuma H (2009). Gene Expr. Patterns.

[14] Peterfi Z, Donko A, Orient A, Sum A, Prokai A, Molnar B, Vereb Z, Rajnavölgyi E, Kovacs KJ, Müller V, Szabo AJ, Geiszt M (2009). Am. J. Pathol.

[15] Gotenstein JR, Swale RE, Fukuda T, Wu Z, Giurumescu CA, Goncharov A, Jin Y, Chisholm AD (2010). Development.

[16] Shi R, Hu C, Yuan Q, Yang T, Peng J, Li Y, Bai Y, Cao Z, Cheng G, Zhang G (2011). Cardiovasc. Res.

[17] Bai Y-P, Hu C-P, Yuan Q, Peng J, Shi R-Z, Yang T-L, Cao Z-H, Li Y-J, Cheng G, Zhang G-G (2011). Free Radical Biol. Med.

[18] Khan K, Rudkin A, Parry DA, Burdon KP, McKibbin M, Logan CV, Abdelhamed ZI, Muecke JS, Fernandez-Fuentes N, Laurie KJ, Shires M, Fogarty R, Carr IM, Poulter JA, Morgan JE, Mohamed MD, Jafri H, Raashid Y, Meng N, Piseth H, Toomes C, Casson RJ, Taylor GR, Hammerton M, Sheridan E, Johnson CA, Inglehearn CF, Craig JE, Ali M (2011). Am. J. Hum. Genet.

[19] Bella J, Hindle KL, McEwan PA, Lovell SC (2008). Cell. Mol. Life Sci.

[20] Kim HM, Oh SC, Lim KJ, Kasamatsu J, Heo JY, Park BS, Lee H, Yoo OJ, Kasahara M, Lee J-O (2007). J. Biol. Chem.

[21] Kobe B, Kajava AV (2001). Curr. Opin. Struct. Biol.

[22] Enkhbayar P, Kamiya M, Osaki M, Matsumoto T, Matsushima N (2004). Proteins: Struct., Funct., Bioinf.

[23] Halaby DM, Mornon JP (1998). J. Mol. Evol.

[24] Barclay AN (2003). Semin. Immunol.

[25] Kaas Q, Ehrenmann F, Lefranc MP (2007). Brief. Funct. Genomics Proteomics.

[26] von Castelmur E, Marino M, Svergun DI, Kreplak L, Ucurum-Fotiadis Z, Konarev PV, Urzhumtsev A, Labeit D, Labeit S, Mayans O (2008). Proc. Natl. Acad. Sci. U. S.A.

[27] O'Leary JM, Hamilton JM, Deane CM, Valeyev NV, Sandell LJ, Downing AK (2004). J. Biol. Chem.

[28] Sadler JE (2009). J. Thromb. Haemostasis.

[29] Holbourn KP, Perbal B, Ravi Acharya K (2009). J. Cell. Commun. Signal.

[30] Zhang J-L, Qiu L-Y, Kotzsch A, Weidauer S, Patterson L, Hammerschmidt M, Sebald W, Mueller TD (2008). Dev. Cell.

[31] Hansson M, Olsson L, Nauseef WM (2006). Arch. Biochem. Biophys.

[32] Wang J, Slungaard A (2006). Arch. Biochem. Biophys.

[33] Ihalin R, Loimaranta V, Tenuovo J (2006). Arch. Biochem. Biophys.

[34] Rees MD, Kennet EC, Whitelock JM, Davies MJ (2008). Free Radical Biol. Med.

[35] Wang Y, Rosen H, Madtes DK, Shao B, Marten TR, Heinecke JW, Fu X (2007). J. Biol. Chem.

[36] Heinecke JW, Li W, 3rd HLDaehnke,, Goldstein JA (1993). J. Biol. Chem.

[37] Johansson MW, Lind MI, Holmblad T, Thornqvist PO, Soderhall K (1995). Biochem. Biophys. Res. Commun.

[38] Johansson MW, Patarroyo M, Oberg F, Siegbahn A, Nilsson K (1997). J. Cell Sci.

[39] Stofanko M, Kwon SY, Badenhorst P (2008). Genetics.

[40] Olofsson B, Page DT (2005). Dev. Biol.

[41] Passardi F, Theiler G, Zamocky M, Cosio C, Rouhier N, Teixera F, Margis-Pinheiro M, Ioannidis V, Penel C, Falquet L, Dunand C (2007). Phytochemistry.

[42] Larkin MA, Blackshields G, Brown NP, Chenna R, McGettigan PA, McWilliam H, Valentin F, Wallace IM, Wilm A, Lopez R, Thompson JD, Gibson TJ, Higgins DG (2007). Bioinformatics.

[43] Nicholas KB, Nicholas HB

[44] Tamura K, Dudley J, Nei M, Kumar S (2007). Mol. Biol. Evol.

[45] McGuffin LJ, Bryson K, Jones DT (2000). Bioinformatics.

[46] Lambert C, Léonard N, Bolle XDe, Depiereux E (2002). Bioinformatics.

